# Potential Function of Testicular MicroRNAs in Heat-Stress-Induced Spermatogenesis Disorders

**DOI:** 10.3390/ijms24108809

**Published:** 2023-05-16

**Authors:** Mailin Gan, Yunhong Jing, Zhongwei Xie, Jianfeng Ma, Lei Chen, Shunhua Zhang, Ye Zhao, Lili Niu, Yan Wang, Xuewei Li, Li Zhu, Linyuan Shen

**Affiliations:** 1 Key Laboratory of Livestock and Poultry Multi-Omics, College of Animal and Technology, Sichuan Agricultural University, Chengdu 611130, China; ganmailin@sicau.edu.cn (M.G.);; 2 Farm Animal Genetic Resource Exploration and Innovation Key Laboratory of Sichuan Province, Sichuan Agricultural University, Chengdu 611130, China

**Keywords:** heat stress, testicular atrophy, microRNAs, spermatogenesis disorder, infertility

## Abstract

Spermatogenesis is temperature-dependent, and the increase in testicular temperature seriously affects mammalian spermatogenesis and semen quality. In this study, the testicular heat stress model of mice was made with a 43 °C water bath for 25 min, and the effects of heat stress on semen quality and spermatogenesis-related regulators were analyzed. On the 7th day after heat stress, testis weight shrank to 68.45% and sperm density dropped to 33.20%. High-throughput sequencing analysis showed that 98 microRNAs (miRNAs) and 369 mRNAs were down-regulated, while 77 miRNAs and 1424 mRNAs were up-regulated after heat stress. Through gene ontology (GO) analysis of differentially expressed genes and miRNA–mRNA co-expression networks, it was found that heat stress may be involved in the regulation of testicular atrophy and spermatogenesis disorders by affecting cell meiosis process and cell cycle. In addition, through functional enrichment analysis, co-expression regulatory network, correlation analysis and in vitro experiment, it was found that miR-143-3p may be a representative potential key regulatory factor affecting spermatogenesis under heat stress. In summary, our results enrich the understanding of miRNAs in testicular heat stress and provide a reference for the prevention and treatment of heat-stress-induced spermatogenesis disorders.

## 1. Introduction

Infertility has become a global health problem, with 48 million couples and 186 million people suffering from infertility worldwide, according to data from the World Health Organization [1]. Male infertility accounts for about 50% of infertility [2]. The decrease in the number of sperm in semen and the abnormality of sperm movement and morphology are the main reasons for male infertility [3]. A large number of studies have shown that the fertility of men around the world is declining in a “cliff” manner [4]. From 1973 to 2018, the global average sperm number of men decreased by 62% and the sperm concentration decreased by 41.5% [5]. A decline in semen quality was also observed in dogs [6], bulls [7] and stallions [8]. After entering the 21st century, the declining trend of the quantity and quality of male semen is still intensifying, which has caused people to worry about the reasons for the decline in male fertility [3].

The global warming caused by the greenhouse effect is considered to be an important factor affecting the decline in global male semen quality [9,10]. Mammalian sperm is generated in the testis. The temperature of the testis is lower than the core temperature, so the testis is extremely sensitive to the high temperature in the environment [11]. Once testicular heat stress occurs, the sperm quality will be damaged, increasing the risk of infertility [12]. It has been found that the sperm density of men in summer is about 70% of that in winter [13]. The testicles of mice were bathed in water at 40 °C for 20 min, which resulted in a decrease in germ cell proliferation and a decrease in circulating testosterone [14]. Acute heat stress (43 °C, 0.5 h) can reduce the cell viability of porcine immature Sertoli cells (iSCs) cultured in vitro [15]. The changes in gene expression can be detected in mouse testes at 4 h after a single heat shock at 43 °C for 20 min and the weight of testes decreases significantly, which still cannot be recovered on the 68th day [16]. These results indicate that heat stress will have adverse effects on spermatogenic cells and spermatogenic auxiliary cells.

Epigenetic changes are an important part of gametogenesis and related reproductive diseases [17]. As an important epigenetic regulator, noncoding RNA (ncRNA) has been widely reported to participate in testicular development and spermatogenesis [18,19]. MicroRNAs have become one of the most reported ncRNAs due to their high conservatism and stability [20,21]. With the frequent occurrence of extreme weather, the probability of experiencing acute and chronic heat stress in daily life is also increasing, and poor lifestyle habits (such as prolonged sitting, hot baths, tight pants, high-temperature sunbathing, etc.) further exacerbate the risk of people being exposed to environmental heat stress [5]. Male hot baths are a common testicular heat stress event, a hot bath of more than 30 min can significantly reduce rat fertility within one month, during which there are also a certain proportion of sperm with different degrees of damage in the epididymis [22]. A previous study reported the changes in the expression of miRNAs in testes at the early stage (6 h) after heat stress and analyzed the effect of differentially expressed miRNAs at the early stage of heat stress on apoptosis [23]. Related in vitro studies also focused on the early stage after heat stress injury. However, the spermatogenesis process is complex and long, and the overall expression and regulation network of miRNAs in heat-stress-induced testicular atrophy and spermatogenesis disorder is still unclear. In this study, we aimed to find miRNAs that play an important role in spermatogenesis disorder induced by heat stress through mining and analysis of public data combined with preliminary experimental verification, so as to provide reference for the prevention and treatment of testicular heat stress injury.

## 2. Materials and Methods

### 2.1. Animals and Treatment

A total of 32 (33.83 ± 2.19 g) three-month-old male ICR mice were used in this study (CHENGDU DOSSY EXPERIMENTAL ANIMALS CO., LTD., Chengdu, China). Each mouse was housed in a single cage, all mice lived in the animal room at 22 ± 3 °C, and were allowed to freely contact with water and food. This research was approved by the Ethics Committee of Sichuan Agricultural University (Sichuan, China, No. 20210156). After a week of adaptation, we selected 20 mice (34.03 ± 1.06 g) with the closest body weight to construct a testicular heat stress model and the remaining 12 mice (33.49 ± 3.38 g) were used to analyze the correlation between semen indicators.

These 20 mice with similar body weight were randomly divided into control (NC) group (6 mice) and heat stress (HS) group (3d: 4 mice; 7d: 6 mice; 10d: 4 mice). Referencing previous studies to establish a testicular heat stress model, firstly, the mice were anesthetized with 0.01 mL/g of 5% chloral hydrate, and then the mouse scrotum was placed in a water bath at 33 °C (NC group) or 43 °C (HS group) for 25 min. After the water bath, the mice were wiped dry and fed in a normal environment until the end of the experiment [24].

### 2.2. Thermal Imaging and Semen Quality Analysis

The anesthetized mice (0.01 mL/g of 5% chloral hydrate) lay flat on the test bench and were photographed using a thermal imager (850 L, FOTRIC, Shanghai, China). At the end of the experiment, the sperm was washed out from the epididymis of mice with phosphate buffer solution (PBS) by referring to previous studies [25]. The semen quality of mice was analyzed using an automated sperm count and analysis system (AndroVision, Minitube, Tiefenbach, Germany).

### 2.3. Data Analysis

This study uses multiple datasets from the Gene Expression Omnibus (GEO) database (https://www.ncbi.nlm.nih.gov/gds, accessed on 1 September 2022). Sequencing data of miRNAs in testes of mice after heat stress (GSE165697: GSM5048341, GSM5048342, GSM5048343, GSM5048344, GSM5048345, GSM5048346 by Meng et al.). Sequencing data of mRNAs in testes of mice after heat stress (GSE165696: GSM5048335, GSM5048336, GSM5048337, GSM5048338, GSM5048339, GSM5048340 by Meng et al.). Germ cells and reproductive helper cell miRNAs data (GSE125302: GSM3568659, GSM3568660, GSM3568661, GSM3568662, GSM3568663, GSM3568664, GSM3568665, GSM3568666, GSM3568667, GSM3568668, GSM3568669, GSM3568670 by Chen et al.). Germ cells and reproductive helper cell mRNAs data (GSE125303: GSM3568680, GSM3568681, GSM3568682, GSM3568683, GSM3568684, GSM3568685, GSM3568686, GSM3568687, GSM3568688, GSM3568690, GSM3568691 by Chen et al.). Using collagenase digestion and Percoll density gradient separation, primary cells were isolated from 3-month-old mouse testes [26]. Azoospermia-related miRNAs (http://www.cuilab.cn/hmdd/, accessed on 3 September 2022). Based on the count values of each transcript, R4.0.5 software and EdgeR software packages were used to perform differential expression analysis on different transcripts at different stages. For all miRNA and mRNA, according to the relative expression level, when *p* ≤ 0.05 and fold change ≥ 2 were simultaneously met, they were considered differentially expressed RNA. Differential expression of RNA was used for further target gene prediction and functional enrichment analysis.

### 2.4. Target Gene Prediction and miRNA mRNA Network Construction

TargetScan [27] and miRanda [28] were used to predict target genes of miRNA. The online analysis platform (https://www.bioinformatics.com.cn/, accessed on 20 September 2022) was used to perform GO and KEGG function enrichment analysis on target genes of miRNAs and mRNA. The online analysis platform is also used for production of volcano maps and heat maps, gene set enrichment analysis (GSEA), principal component analysis (PCA), and differential expression mRNA chromosome position labeling. The mRNA in the miRNA mRNA network originates from the intersection of differentially expressed miRNA target genes and differentially expressed mRNA. Based on the correspondence between miRNA and target genes, the degree value (lattice width) was calculated using Cytoscape 3.6.0 and GO analysis was performed on the core genes using an online platform. Finally, Adobe Illustrator CS6 was used for graphic adjustment.

### 2.5. Cell Culture

The Sertoli cell line TM4 was purchased from Shanghai Xuanya Biotechnology Co., Ltd. (Shanghai, China). TM4 cells were cultured in DMEM/F12 medium (Meilunbio, Dalian, China) supplemented with 2.5% fetal bovine serum (FBS; Gibco, Carlsbad, CA, USA), 5% horse serum (HS; Gibco) and 1% penicillin/streptomycin (Biyuntian Company, shanghai, China) at 37 °C in 5% CO_2_. The miR-143-3p mimic and mimic control (GenePharma, Shanghai, China) were transfected into the TM4 cells using Lipofectamine 3000 (Invitrogen, Guangzhou, China) according to the manufacturer’s instructions. TM4 cells were harvested at 24 h after transfection and total RNA was extracted. Cells were incubated with an EdU reagent for 2 h (Ribobio, Guangzhou, China) at 24 h after transfection. The EdU staining assay was performed according to the manufacturer’s instructions.

### 2.6. RT-q PCR

About 30 mg of testicular tissue was fully dissolved by 1 mL of Trizol (TaKaRa, Dalian, China) and total RNA was extracted according to the instructions of the kit. Then, the total RNA was reverse transcribed into cDNA, RT-qPCR was performed using SYBR Premix Ex Taq kit (TaKaRa), and the relative expression of mRNA (ACTB as internal reference gene) and miRNA (U6 as internal reference gene) was calculated using 2^−ΔΔCT^ method [29].

### 2.7. Statistical Analysis

Data wrangling and statistical analysis were performed using WPS Office and SPSS 22.0. Data are presented as means ± standard deviation (SD). Differences in phenotypic index and gene expression were determined between two groups using Student’s *t*-test or one-way ANOVA. *p* ≤ 0.05 is considered statistically significant.

## 3. Results

### 3.1. Testicular Heat Stress Leads to Testicular Atrophy and Reduced Spermatogenesis

**Figure 1:**
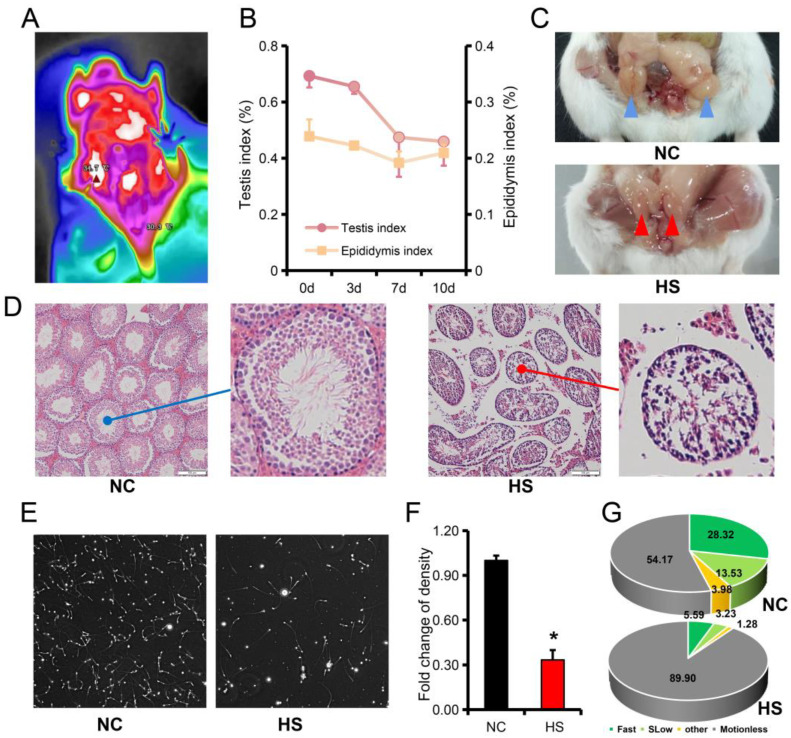
Heat stress leads to reduced spermatogenesis. (**A**) Core body temperature and testicular temperature of mice. (**B**) Changes in testis and epididymis index in mice after heat stress (25 min at 43 °C). (**C**) Morphological characteristics of the testes and epididymis of mice on the 7th day after heat stress. (**D**) Hematoxylin eosin staining of testicular tissue sections in mice after heat stress. (**E**,**F**) Morphological characteristics (**E**) and quantity statistics (**F**) of sperm in mice on the 7th day after heat stress. (**G**) Analysis of sperm motility of mice on the 7th day after heat stress. Results are presented as “means ± SD”. *n* = 3. * *p* < 0.05.

The thermogram showed that the testicular temperature of the mice was about 4 °C lower than the core temperature (Figure 1A). The testis of mice were bathed in water at 43 °C for 25 min to establish a heat stress model. It was found that the testis and epididymis of mice continued to atrophy after heat stress (Figure 1B). On the 7th day after heat stress, the testis atrophied to 68.45% and the epididymis atrophied to 80.28% (Figure 1B,C). Testicular tissue sections showed atrophy of the seminiferous tubules and an increase in the gap between the seminiferous tubules after heat stress (Figure 1D). The sperm density also decreased to 33.20% of the NC group (Figure 1E,F, Videos S1–S2). Further analysis of sperm movement trajectory showed that progressive motility sperm after heat stress was only 21.08% of NC group (Figure 1G).

### 3.2. Abnormal Cell Cycle of Testicular Tissue after Testicular Heat Stress

**Figure 2:**
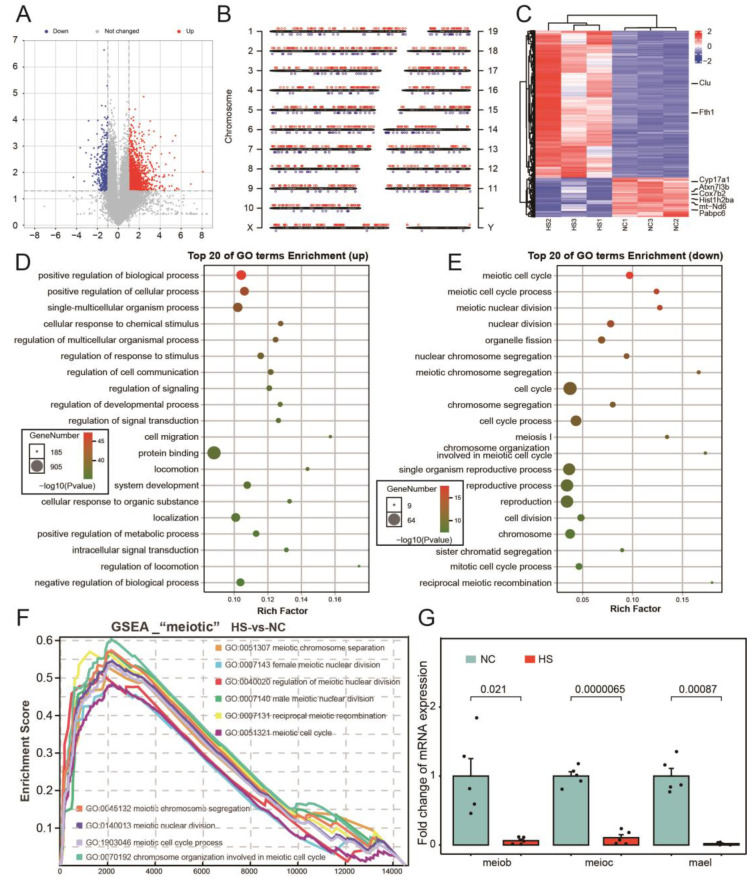
Testicular heat stress leads to abnormal meiosis. (**A**) Volcanic map shows genes differentially expressed in testes of HS group and NC group. (**B**) The distribution of differentially expressed genes on chromosomes. (**C**) The heat map shows the clustering results of samples based on differentially expressed genes; the labeled mRNAs belong to high expression mRNAs (Top 500 highly expressed mRNAs). (**D**,**E**) Gene ontology (GO) analysis results of up-regulated genes (**D**) and down-regulated genes (**E**) after heat stress. (**F**) Gene set enrichment analysis (GSEA) of biological processes related to “meiotic”. (**G**) The relative expression of meiob, meioc and mael in the testis of mice after heat stress, *n* = 5.

The trend of testicular weight changes (with a fast atrophy rate in the 3rd to 7th days and slowed down in the 7th to 10th days) suggests that the 7th day after heat stress is a time point worth studying (Figure 1B). In addition, the 7th day after testicular heat stress was also a commonly used time point in previous studies [30,31]. High-throughput sequencing results showed that 1422 genes were up-regulated and 369 genes were down-regulated in testicular tissues after heat stress (Figure 2A). The most up-regulated genes were distributed on chromosome 11, and the most down-regulated genes were distributed on chromosome 14 (Figure 2B). The heat map shows the differential genes and the good clustering of NC group and HS group into two branches, suggesting a good repeatability between the two groups of samples (Figure 2C). After heat stress, the up-regulated genes are mainly involved in positive regulation of biological process, positive regulation of cellular process and single-multicellular organism process, while the down-regulated genes are mainly involved in meiotic cell cycle, meiotic cell cycle process and meiotic nuclear division (Figure 2D,E). Through gene set enrichment analysis (GSEA), it was found that biological processes related to “meiotic” were significantly lower in HS group than in NC group (Figure 2F). Then, we used RT-qPCR to verify the relative expression levels of meiob, meioc, and mael, which are marker genes related to meiosis (Figure 2G).

### 3.3. Testicular Heat Stress Changes the Expression Profile of miRNAs in Testicular Tissue

**Figure 3:**
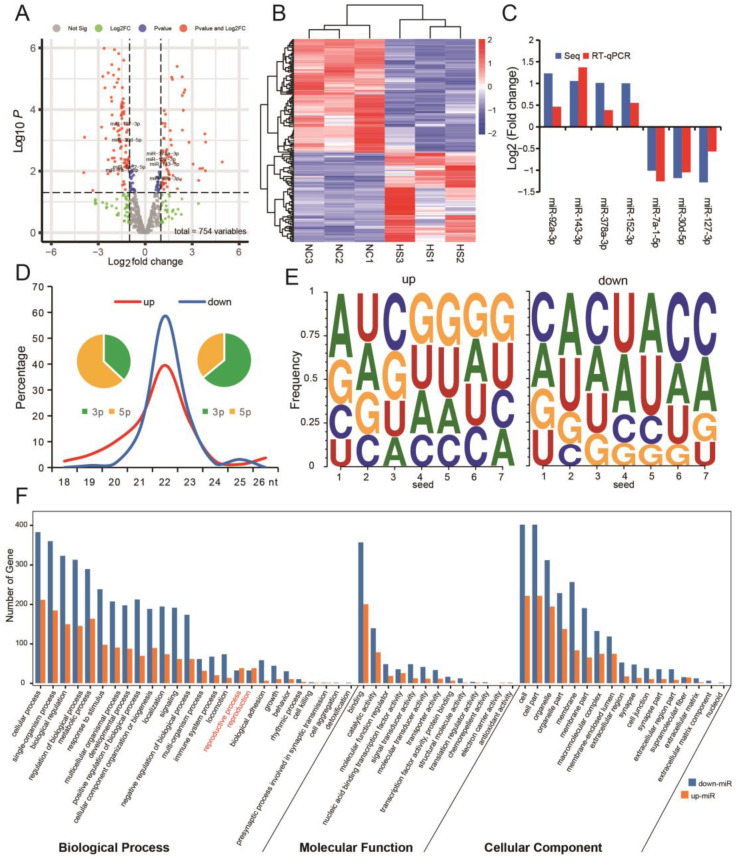
Expression profile of miRNAs in heat-stressed testis. (**A**) Volcanic map shows miRNAs differentially expressed in testes of HS group and NC group, the labeled miRNAs belong to high expression miRNAs (top 100 highly expressed miRNAs). (**B**) The heat map shows the clustering results of samples based on differentially expressed miRNAs. (**C**) Detection of miRNA expression by RT-qPCR (top 100 highly expressed miRNAs and |log_2_ fold change| ≥ 1), *n* = 3. (**D**) Sequence characteristics of differentially expressed miRNAs. (**E**) Characteristics of seed sequences of differentially expressed miRNAs. (**F**) GO analysis results of target genes of differentially expressed miRNAs.

A total of 77 miRNAs were up-regulated and 98 miRNAs were down-regulated in heat-stressed testes (Figure 3A). Clustering based on differential miRNAs can group the samples in HS group and NC group into two independent branches, indicating that the two groups of samples have good repeatability (Figure 3B). In addition, RT-qPCR results of differentially expressed miRNAs also have the same trend as sequencing results (Figure 3C). Further analysis showed that the main type of up-miRNAs was 3p and the main type of down-miRNAs was 5p (Figure 3D). The seed sequence characteristics of differentially expressed miRNAs also showed different rules (Figure 3E). GO analysis of target genes of differentially expressed miRNAs found that up-regulated miRNAs enriched more genes than down-regulated miRNAs only during reproduction (Figure 3F). These results suggest that the up-regulated miRNAs after heat stress may have important biological significance in the reproductive process.

### 3.4. Differential miRNA and mRNA Co-Expression Networks in Testicular Tissue of Normal and Heat-Stressed Mice

**Figure 4:**
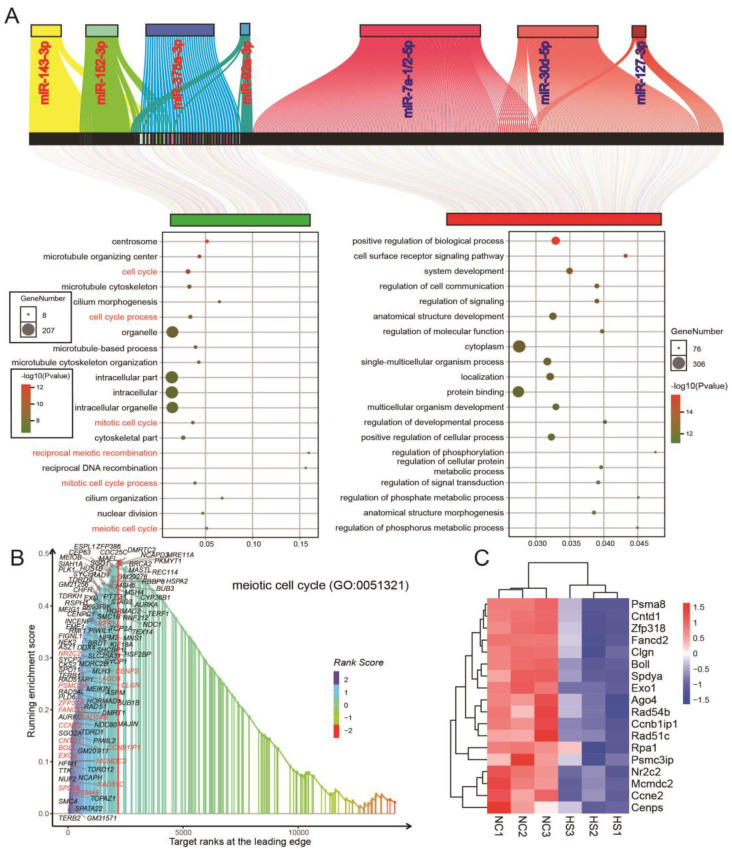
Co-expression regulatory network of miRNA–mRNA in mice testis. (**A**) The sankey diagram shows the co-expression network and GO analysis results of differentially expressed miRNA and mRNA after heat stress. (**B**) GSEA of “meiotic cell cycle”. (**C**) Cluster analysis of differential expression of mRNAs in “meiotic cell cycle” (red font in (**B**)).

Further, we constructed a miRNA-mRNA co-expression network based on highly and differentially expressed miRNAs (Figure 4A). The co-expression network showed that up-miRNAs and down-mRNAs were mainly involved in the centrosome, microtubule organizing center and cell cycle, while down-miRNAs and up-mRNAs were mainly involved in positive regulation of biological process, cell surface receptor signaling pathway and system development (Figure 4A). These results are similar to those of differential mRNA and miRNAs previously analyzed separately (Figure 2D,E and Figure 3F). We selected the “meiotic cell cycle” (which is the most significant biological process among differentially expressed genes) for GSEA analysis and found that the mRNAs down-regulated after heat stress in the co-expression network were all located on the left side of the peak value of the enrich score and these genes can group samples of HS group and NC group into two independent branches (Figure 4B,C).

### 3.5. Expression Characteristics of Differentially Expressed miRNAs Induced by Heat Stress in Different Cell Groups of Mice Testis

**Figure 5:**
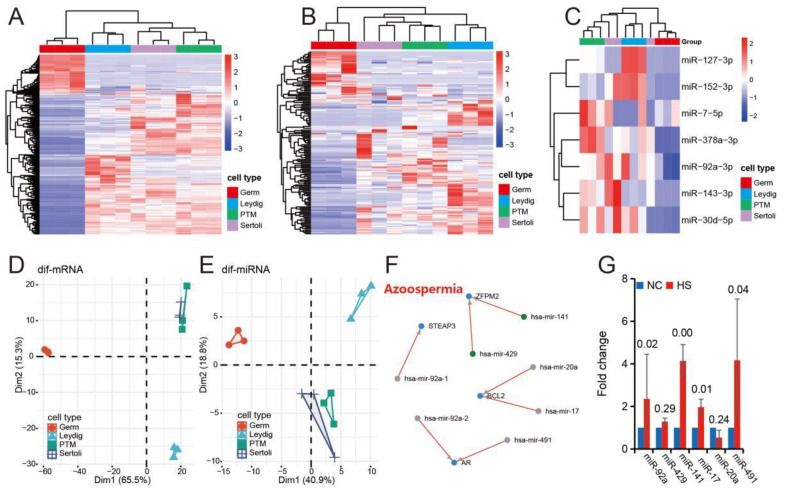
Cell expression characteristics of differentially expressed miRNAs. (**A**,**B**) The heat map shows the clustering analysis results of different cell group samples based on differentially expressed mRNA (**A**) and miRNAs (**B**). (**C**) Clustering analysis results of different cell group samples based on miR-127-3p, miR-152-3p, miR-7-5p, miR-378a-3p, miR-92a-3p, miR-143-3p and miR-30d-5p (same as Figure 3C, top 100 highly expressed miRNAs and |log2 fold change| ≥ 1). (**D**,**E**) The scatter plot shows the results of principal component analysis (PCA) of samples from different cell groups based on differentially expressed mRNA (**D**) and miRNAs (**E**). (**F**,**G**) miRNAs related to azoospermia ((**F**), data from Human microRNA Disease Database (HMDD, http://www.cuilab.cn/hmdd/, accessed on 28 November 2022) and their expression (**G**) in heat-stressed testis, *n* = 3.

Cluster analysis of major cell groups in testis based on differentially expressed mRNAs (Figure 2A) and differentially expressed miRNAs (Figure 3A) after heat stress shows that mRNAs and miRNAs have similar expression characteristics of different cell groups (Figure 5A,B). Clustering with the first 100 differentially expressed miRNAs can also cluster germ cells, Sertoli cells and peritubular muscle-like cells into different branches, indicating that these miRNAs can better represent all differentially expressed miRNAs (Figure 5C). The results of principal component analysis (PCA) are similar to those of clustering in heat map (Figure 5D,E). Further, we analyzed the expression pattern of miRNAs that may be related to azoospermia after heat stress in the testes and found that, except miR-429 and miR-20a, other miRNAs showed significant differential expression between heat-stressed testes and normal testes (Figure 5F,G).

### 3.6. miR-143-3p Can Be Used as a Representative Potential Biomarker of Testicular Heat Stress

**Figure 6:**
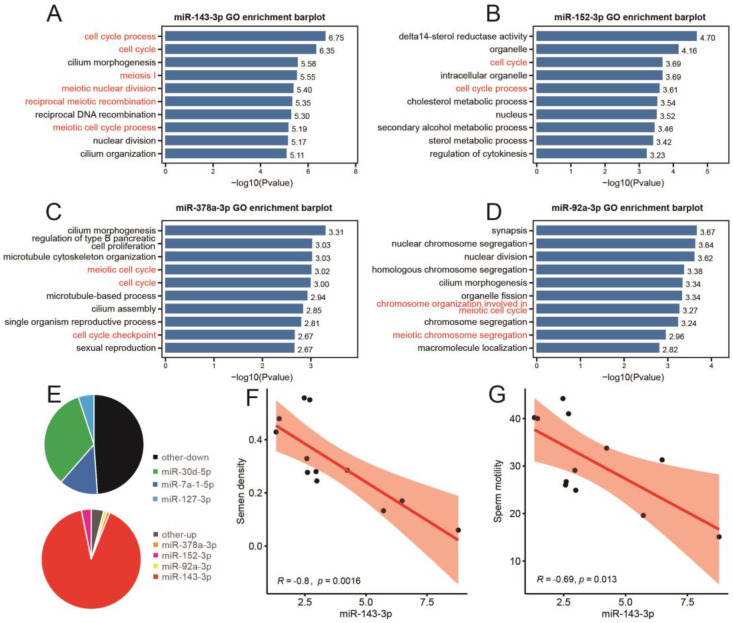
miR-143-3p can be used as a potential marker of testicular heat stress. (**A**–**D**) GO analysis results of target genes of miR-143-3p (**A**), miR-152-3p (**B**), miR-378a-3p (**C**) and miR-92a-3p (**D**). (**E**) Composition of differentially expressed miRNAs. (**F**,**G**) Correlation between the relative expression of miR-143-3p and sperm density (**F**) and sperm motility (**G**), *n* = 12.

Based on the above research results (Figure 3C and Figure 5C), we preliminarily identified miR-143-3p, miR-152-3p, miR-378a-3p and miR-92a-3p as potential markers involved in testicular heat-stress-induced spermatogenesis disorder. GO analysis showed that the target genes of miR-143-3p were mainly enriched in cell cycle process, cell cycle, cilia morphogenesis, meiosis I and medical nuclear division (Figure 6A). The target genes of miR-152-3p are mainly enriched in delta14 sterol reductase activity, organelle, cell cycle, intelligent organelle and cell cycle process (Figure 6B). The target genes of miR-378a-3p are mainly enriched in cilium morphogenesis, regulation of type B pancreatic cell promotion, microtubule cytoskeleton organization, medical cell cycle and cell cycle (Figure 6C). The target genes of miR-92a-3p are mainly enriched in synapsis, nuclear chromosomal aggregation, nuclear division, homologous chromosomal aggregation and cilium morphogenesis (Figure 6D). Further analysis of the proportion of each differentially expressed miRNA showed that miR-143-3p accounted for 90.78% of all up-regulated miRNAs after heat stress (Figure 6E). In addition, the expression of miR-143-3p in testis was negatively correlated with sperm density and sperm motility (Figure 6F,G).

**Figure 7:**
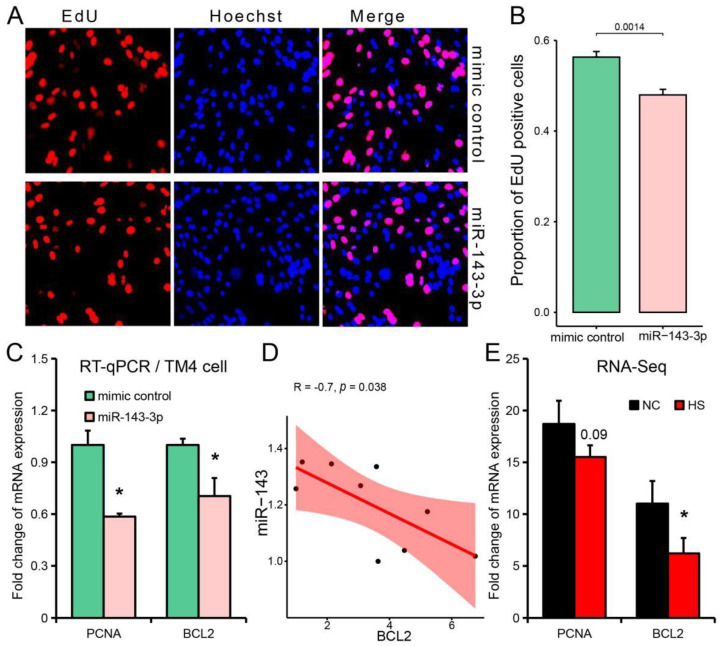
miR-143-3p inhibits Sertoli cell proliferation. (**A**,**B**) EdU staining (**A**) and positive cell count (**B**) after transfection of miR-143-3p mimic into Sertoli cells (TM4 cell line), *n* = 3. (**C**) Relative expression levels of PCNA and BCL2 after transfection of miR-143-3p mimic into Sertoli cells, *n* = 3. (**D**) Correlation between the expression level of miR-143-3p and BCL2 in Sertoli cells, *n* = 9. (**E**) The expression level (RNA-seq) of PCNA and BCL2 in testis under heat stress. Results are presented as “means ± SD”, *n* = 3. * *p* < 0.05.

Since the expression level of miR-143-3p was the highest in Sertoli cells (Figure 5C), we further explored the influence of miR-143-3p on proliferation in Sertoli cells (TM4 cell lines were used in this study). The number of EdU (5-ethynyl-20-deoxyuridine) positive cells in Sertoli cells was significantly decreased after miR-143-3p mimic transfection (Figure 7A,B). Meanwhile, RT-qPCR results showed that transfected miR-143-3p mimic into Sertoli cells significantly inhibited the expression of proliferating cell nuclear antigen (PCNA) [32] and B cell leukemia/lymphoma 2 (BCL2) [33], which are markers of azoospermia (Figure 5F) and cell proliferation (Figure 7C). The expression level of miR-143-3p was also negatively correlated with the expression level of BCL2 (a target gene of miR-143-3p [34,35]) in TM4 cell lines (Figure 7D). In addition, the expression of BCL2 was also significantly decreased in heat-stressed testis (Figure 7E).

## 4. Discussion

Male infertility has become a common problem in the world [36] and a decrease in sperm quality is the main cause of male infertility [37]. Spermatogenesis is a complex and long process. Sperm is formed in the seminiferous tubules of the testis and develops from spermatogonia [38]. Type A undifferentiated spermatogonium cells enter the proliferating pool and one of the daughter cells returns to the spermatogonal stem cell pool, while the other daughter cell (A1 spermatogonium) continues to differentiate and divide once, producing two type A2 spermatogonium cells. Two A2-type cells divide to produce 4 A3, 8 A4, 16 intermediate spermatogonia, 32 B-type spermatogonia, and finally form 64 primary spermatocytes. One tetraploid primary spermatocyte produces two diploid secondary spermatocytes and, ultimately, four haploid sperm cells [39]. In mice, a seminiferous epithelium cycle is about 8.6 days and a complete spermatogenic cycle is about 35 days [40], while, in humans, a seminiferous epithelium cycle is about 16 days and a complete spermatogenic cycle is about 74 days [41]. It is easy to be affected by environmental factors in the long process of spermatogenesis, which leads to spermatogenesis disorder [42]. In theory, each A1 spermatogonium cell produces 256 sperm but, due to a variety of factors, only 20–30% of mature sperm are formed in mammals [43]. Mammalian sperm is mainly produced in the testis, and the temperature of the testis is usually 2–8 °C lower than the core temperature [44], so the process is extremely sensitive to high temperature [45]. As an epigenetic regulator with excellent conservation and stability, microRNAs have been reported to be involved in spermatogenesis [20,46]. A previous study reported the changes in miRNAs’ expression profile in a short time after testicular heat stress [23], but the spermatogenesis process is long and complex and there is still a lack of comprehensive understanding of miRNA expression changes after testicular heat stress. In this study, the miRNA expression profile of testis after 7 days (about one cycle of spermatogenic epithelium (8.6 days [47])) of heat stress in mice was analyzed, and the miRNAs–mRNAs co-expression network of testis was constructed, providing a reference for the research on testis heat stress.

Spermatozoa carries the task of accurate intergenerational transmission of paternal genomic genetic information, which is an important guarantee for species reproduction and species continuity. Testis are the main site of mammalian spermatogenesis and the only visceral organ located outside the body, which makes spermatogenesis extremely sensitive to the external environment [48]. Since spermatogenesis requires a relatively low-temperature environment, temperature is the most important environmental factor affecting spermatogenesis [49]. The adverse effect of temperature on spermatogenesis is not only manifested in the decline in male semen quality under the background of global warming, but also manifested in the seasonal fluctuation of semen quality [50]. The seasonal decline in mammalian reproductive performance mainly occurs in summer (high-temperature weather), mainly manifested by a decline in semen quality. This pattern of change has been found in humans [51] and large domestic animals (boar [52], bovine [53] and ram [54]). The increase in scrotal temperature will produce testicular heat stress, which will subsequently lead to testicular atrophy and spermatogenesis stagnation, leading to a decrease in sperm number [55,56]. In this study, it was found that the testicles continued to atrophy within 10 days after heat stress, with the fastest atrophy rate in the 3rd to 7th days, and slowed down in the 7th to 10th days. This may be related to the 8.6 days of a seminiferous epithelium cycle in mice [47] and one-time heat stress may mainly damage a seminiferous epithelium cycle. Previous studies have also found that transient heat stress can be recovered after 1–2 complete spermatogenic cycles [57,58]. On the 7th day after heat stress, we observed a decrease in the number of sperm in the epididymis of mice, and the corresponding transcriptional evidence was that a large number of genes down-regulated after heat stress were involved in meiosis and cell cycle processes. The study on bulls also found that the number of head-defective sperm increased and the mitochondrial membrane potential of sperm decreased within 7 days after heat stress [59]. The increase in apoptosis and the decrease in cell proliferation after heat stress are the main causes of testicular atrophy [23], and the decrease in sperm number may be related to the inhibition of spermatogonial stem cell proliferation [60].

Epigenetics mainly studies the interaction between environment and gene and reveals the influence of environment on biological genetics. As a highly conservative and stable epigenetic regulator, miRNAs have been reported to participate in the process of transgenerational inheritance [61]. Therefore, analyzing the changes in miRNAs in heat-stressed testes may provide a reference for coping with the male reproductive crisis caused by global warming. In this study, 77 miRNA expressions were up-regulated and 98 miRNAs were down-regulated after 7 days of testicular heat stress. Interestingly, functional enrichment analysis showed that down-regulated miRNAs dominated most signaling pathways, while up-regulated miRNAs enriched more genes than down-regulated miRNAs during reproduction and the reproductive process. These results suggest that miRNAs up-regulated after testicular heat stress play an important role in reproduction. In addition, this study also found that different cell types in the testis have different mRNA and miRNA expression characteristics, and there are significant differences between germ cells and other reproductive helper cells. Differently and highly expressed miRNAs are mainly overexpressed in reproductive helper cells rather than in germ cells (Figure 5A–C). On the one hand, it may be caused by transcriptional inhibition of sperm cells [62] and, on the other hand, it suggests that reproductive helper cells may play an important regulatory role in maintaining homeostasis in the internal environment.

**Figure 8:**
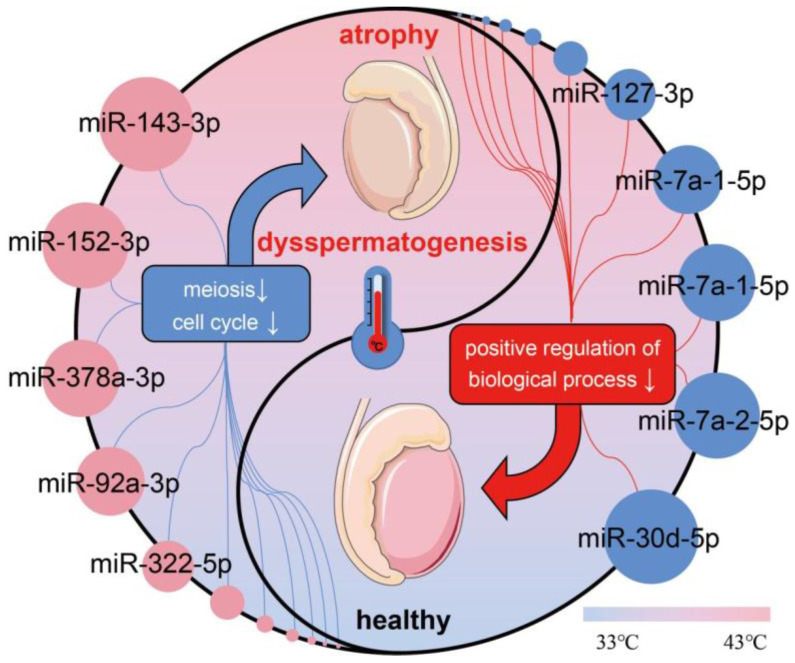
miRNAs that play a potentially important role in testicular heat stress injury.

Further analysis showed that miRNAs differentially expressed after 6h of testicular heat stress, except for miR-3968, were also differentially expressed on the 7th day of heat stress (Table S1). This indicates that there is little difference in differential miRNAs between 6 h and 7 days after heat stress and they have the potential to serve as markers of heat stress damage [23]. miR-423-3p [63], miR-128-3p [64] and miR-423-5p [65] have also been reported to be involved in the regulation of spermatogenesis or animal semen quality. Among them, the research on the mechanism of miR-128-3p is relatively clear. Zou et al. found that the down-regulated expression of miR-128-3p can induce apoptosis of spermatid cells and inhibit their proliferation by promoting MAPK14 phosphorylation [66]. In addition, the comparative analysis of azoospermia-related miRNAs in Human microRNA Disease Database (HMDD) also found that there were differences between the normal and the heat stress group, except for miR-429 and miR-20a [67]. These results suggest that these miRNAs with the same expression pattern have the potential to become biomarkers of testicular heat stress and may be a target for preventing and treating heat stress damages. Co-expression regulatory network and functional enrichment analysis showed that down-regulated miRNAs were mainly involved in the positive regulation of biological processes, cell surface receptor signaling pathway and system development, while up-regulated miRNAs were mainly involved in the centrosome, microtubule organizing center and cell cycle. These suggest that up-regulation of miRNAs is more closely related to spermatogenesis (Figure 8). We further predicted the function of the differentially expressed miRNAs in the top 100 and found that these miRNAs (miR-143-3p, miR-152-3p, miR-378a-3p and miR-92a-3p) were related to cell cycle or meiosis. miR-143-3p [68], miR-152-3p [69], miR-378a-3p [70] and miR-92a-3p [71,72] have also been reported to be involved in the regulation of spermatogenesis or semen quality. It is interesting that there is no significant difference in the miRNAs (miR-20, miR-21 and miR-106a) involved in the self-renewal of spermatogonial stem cells [46], indicating that testicular heat stress under the conditions of this study has little effect on spermatogonial stem cells and they have good recovery ability after testicular heat stress injury. In addition, the miRNAs differentially expressed after heat stress found in the study on the miRNAs of bull spermatozoa and extravesicles of sperm plasma cells are different from those in our study [73]. The reason for this difference may be that the expression characteristics of miRNAs in extracellular vesicles are different from those in testicular tissues, because the results in this study are highly consistent with those in testicular heat stress 6 h later [23]. In addition to meiosis, cell cycle and apoptosis, Xu et al. also found that inflammatory factors in supporting cells were up-regulated after heat stress and the expression of inflammation-related miRNAs (miR-132, miR-431 and miR-543) was decreased [74]. In our study, we also found a decrease in the expression level of miR-543, but there was no significant change in miR-132 and miR-431. This may be due to the testicular tissue we used and the cells used in Xu et al.’s study, but the preliminary results indicate that miR-543 has further research value.

It is worth noting that this study found that the expression level of miR-143-3p accounted for 90.78% of all up-regulated miRNAs, suggesting that miR-143-3p has an important function in testis under heat stress. Correspondingly, the expression level of miR-143-3p in testis was significantly negatively correlated with sperm density and motility. In vitro experiments showed that miR-143-3p could inhibit the proliferation of Sertoli cells. In previous studies, it was also found that miR-143-3p, as a multifunctional miRNA, participates in normal development and disease occurrence [75,76]. miR-143 can inhibit the proliferation of smooth muscle cells by targeting Elk-1 (Ets-like protein 1) and Klf4 (Kruppel-like factor4) [77], miR-143-3p promotes cardiomyocyte proliferation in mice with myocardial infarction through Yap/Ctnnd1 [78], miR-143 inhibits the proliferation of leukemia cells by inhibiting the expression of KAT6A [79], and miR-143 can inhibit the proliferation of breast cancer cells by regulating ERBB3 [80]. These results suggest that miR-143 has an important regulatory role in regulating cell proliferation. In this study, we found that miR-143 can inhibit the proliferation of TM4 cells and down-regulate BCL2, which is the target gene of miR-143 [34,35]. Interestingly, previous studies have shown that protein extraction from the Bcl-2 family is crucial for male germ cell homeostasis [81,82], and BCL2 has also been found to be positively correlated with bull fertility [83]. In addition, differently expressed miRNAs are highly expressed in germ helper cells rather than germ cells (Figure 5C), suggesting that these miRNAs may participate in cross-cell communication in some way (such as exosome), which will be content worthy of attention in the future. In the future, we will focus on the role of heat-stress-induced up-regulation of miRNAs in testicular atrophy and spermatogenesis and carry out more detailed molecular function verification research in vivo and in vitro.

## 5. Conclusions

In this study, the acute testicular heat stress model of mice was constructed by 43 °C hot water bath and it was found that heat stress can lead to testicular atrophy and sperm reduction. On the 7th day after heat stress, 98 miRNAs were down-regulated and 77 miRNAs were up-regulated in testicular tissues. Functional enrichment analysis showed that up-regulated miRNAs are mainly involved in reproduction and reproductive processes through influencing meiosis and cell cycle. In addition, miR-143-3p has important potential as a target for the prevention and treatment of testicular heat stress due to its high proportion (90.78%) in differentially expressed miRNAs and its significant negative correlation with sperm density and motility. In conclusion, this study enriched people’s understanding of the response of noncoding RNA to heat stress in the testis.

## Data Availability

All data involved in this study are available on contact with corresponding authors.

## Supplementary Materials

The following supporting information can be downloaded at: https://www.mdpi.com/article/10.3390/ijms24108809/s1.
